# Co-inhibition of TIGIT and PD-1/PD-L1 in Cancer Immunotherapy: Mechanisms and Clinical Trials

**DOI:** 10.1186/s12943-023-01800-3

**Published:** 2023-06-08

**Authors:** Xianjing Chu, Wentao Tian, Ziqi Wang, Jing Zhang, Rongrong Zhou

**Affiliations:** 1 Department of Oncology, Xiangya Hospital, Central South University, No. 87 Xiangya Road, Kaifu District, Changsha, 410008 China; 2 Present Address: Xiangya Lung Cancer Center, Xiangya Hospital, Central South University, Changsha, 410008 China; 3 National Clinical Research Center for Geriatric Disorders, Xiangya Hospital, Central South University, Changsha, 410008 Hunan Province P.R. China

**Keywords:** Immune checkpoint inhibitors, TIGIT, PD-1, PD-L1, Combined therapy

## Abstract

Over the past decade, immune checkpoint inhibitors (ICIs) have emerged as a revolutionary cancer treatment modality, offering long-lasting responses and survival benefits for a substantial number of cancer patients. However, the response rates to ICIs vary significantly among individuals and cancer types, with a notable proportion of patients exhibiting resistance or showing no response. Therefore, dual ICI combination therapy has been proposed as a potential strategy to address these challenges. One of the targets is TIGIT, an inhibitory receptor associated with T-cell exhaustion. TIGIT has diverse immunosuppressive effects on the cancer immunity cycle, including the inhibition of natural killer cell effector function, suppression of dendritic cell maturation, promotion of macrophage polarization to the M2 phenotype, and differentiation of T cells to regulatory T cells. Furthermore, TIGIT is linked with PD-1 expression, and it can synergize with PD-1/PD-L1 blockade to enhance tumor rejection. Preclinical studies have demonstrated the potential benefits of co-inhibition of TIGIT and PD-1/PD-L1 in enhancing anti-tumor immunity and improving treatment outcomes in several cancer types. Several clinical trials are underway to evaluate the safety and efficacy of TIGIT and PD-1/PD-L1 co-inhibition in various cancer types, and the results are awaited. This review provides an overview of the mechanisms of TIGIT and PD-1/PD-L1 co-inhibition in anti-tumor treatment, summarizes the latest clinical trials investigating this combination therapy, and discusses its prospects. Overall, co-inhibition of TIGIT and PD-1/PD-L1 represents a promising therapeutic approach for cancer treatment that has the potential to improve the outcomes of cancer patients treated with ICIs.

## Introduction

Immune checkpoint inhibitors (ICIs) have significantly advanced cancer treatment by blocking signals that allow cancer cells to evade immune detection, providing durable responses and long-term survival benefits for many cancer patients since the first approval of ipilimumab in 2010 [1]. PD-1/PD-L1 blockades are the most extensively studied ICIs therapy to date, and it has shown that they offered notable survival benefits for metastatic non-small cell lung cancer (NSCLC), improving the median overall survival to 21.9 months [2]. However, response rates can vary across different cancers and individuals, and a significant proportion of patients hardly respond or eventually develop resistance during treatment. For instance, only 20.06% of lung cancer patients are expected to benefit from ICIs, with less than 1.5% of patients experiencing complete responses and around 15% showing partial responses [3]. This is partly due to the complex interplay between cancer cells and the immune system [4–6]. For example, some cancer cells can downregulate molecules that promote T-cell activation, leading to resistance to ICIs [7, 8]. Additionally, factors such as regulatory T cells (Tregs), myeloid-derived suppressor cells (MDSCs), and immunosuppressive cytokines within the tumor microenvironment can inhibit anti-tumor immunity, leading to a predominantly immunosuppressive microenvironment [8–10]. Moreover, no biomarkers currently precisely predict which patients will benefit from ICIs [11–13].

To address these limitations, researchers are exploring combination therapies that target multiple checkpoint molecules or combine ICIs with other treatments such as chemotherapy, radiation therapy, or targeted therapy [14]. One such strategy is dual ICI combination therapy, which targets two inhibitory receptors simultaneously to enhance the anti-tumor immune response. Moreover, dual ICI therapy may provide an opportunity to expand the proportion of patients who respond to immunotherapy and overcome resistance to PD-1/PD-L1 blockades [15, 16]. T cell immunoreceptor with Ig and immunoreceptor tyrosine-based inhibitory motif (ITIM) domains (TIGIT) has emerged as a promising target for co-inhibition with PD-1/PD-L1 in cancer immunotherapy [10]. It has been proven that TIGIT is associated with T-cell exhaustion and immunosuppressive effects across all stages of the cancer immunity cycle [17–21]. Moreover, co-inhibition of TIGIT and PD-1/PD-L1 enhances anti-tumor immunity and improves treatment outcomes in various cancers in preclinical and clinical studies [22–24].

This review provides a comprehensive summary of the roles of TIGIT in cancer immunity, the mechanisms of co-inhibition of TIGIT and PD-1/PD-L1, and the current clinical trials of this combination therapy. Furthermore, we highlight the current challenges of the novel therapeutic strategies and discuss future efforts to make a breakthrough in anti-tumor treatment.

## The central role of TIGIT in the cancer immunotherapy

TIGIT, initially identified in 2009, belongs to the type 1 poliovirus receptor (PVR) and is a member of the nectin family [21, 25]. Typically, TIGIT acts as a co-inhibitory receptor, widely expressed on CD4^+^ T cells, CD8^+^ T cells, and Tregs [1]. Its cytoplasmic region contains an immunoglobulin tyrosine tail (ITT)-like motif and a standard ITIM. The ligands of TIGIT comprise CD155 (PVR or Necl-5), CD113 (PVRL3 or Nectin-3), CD112 (PVRL2 or Nectin-2), and PVRL4 (Nectin-4) [24, 25]. Functionally, TIGIT has been demonstrated to be crucial in inducing immunosuppressive effects in cancer immunotherapy, like CTLA-4 and PD-1/PD-L1 [26–28].

### Direct inhibitory effects of TIGIT in T and NK cells

Natural killer (NK) cells are the main forces of anti-tumor innate immunity, while T cells are those of adaptive immunity, both of which are crucial components of anti-tumor immunity. Previous studies demonstrated that TIGIT was expressed on exhausted TOX^high^ TCF-1^high^ CD8^+^ T cell subsets in both mice and humans and was identified as a marker for T-cell exhaustion [29–31]. *Eomes*, a transcription factor with a key role in CD8^+^ T cell differentiation, by binding to the promoter of *TIGIT*, upregulate its expression [32]. Also, TIGIT^+^ NK cells display weaker anti-tumor cytotoxicity than TIGIT^−^ NK cells [33].

One of the mechanisms by which TIGIT lessens the toxicity of T/NK cells is its intracellular signaling domains. Upon CD155 binding to TIGIT, the ITT-like motif is phosphorylated and binds to Grb2, bringing about the recruitment of SH domain-containing inositol-5-phosphatase (SHIP1) and impeding multiple signaling pathways [28]. SHIP1 is a crucial inhibitor of the phosphatidylinositol 3-kinase (PI3K) signaling, as it hydrolyzes PI(3,4,5)P3, thereby inhibiting kinases containing pleckstrin homology (PH) structural domains, such as Akt, Btk, and phospholipase C-γ [34]. Moreover, premature binding of TIGIT to CD155 hinders phosphorylation of Erk and MEK kinases, which are initiators of the MAPK signaling cascade. Blocking the TIGIT signaling rescues Erk phosphorylation following TIGIT/CD155 binding, and silencing SHIP1 reverses TIGIT/CD155-mediated inhibition, thus restoring cytotoxicity of NK cells [35]. The nuclear factor-κB (NF-κB) pathway also plays a crucial role in the TIGIT/CD155-mediated immunosuppression, as TIGIT inhibition increased p-Erk, p-IκBα, and p-NF-κBP65 levels, and decreased SHIP1 expression in activated T-cell culture [34]. Animal models also suggest that TIGIT, upon binding to and activation by CD155, suppresses PI3K, MAPK, and NF-κB pathways by recruiting SHIP1, resulting in depletion of T and NK cells and less production of interferon-γ [34]. Significantly, either phosphorylation of ITIM (Y227) or ITT-like motif (Y233) triggers TIGIT inhibitory signaling in mice. However, TIGIT/CD155 binding initiates the primary inhibitory signal through the ITT-like motif, and the ITIM motif mediates the following inhibitory signaling in human cell lines [21, 34].

**Fig. 1:**
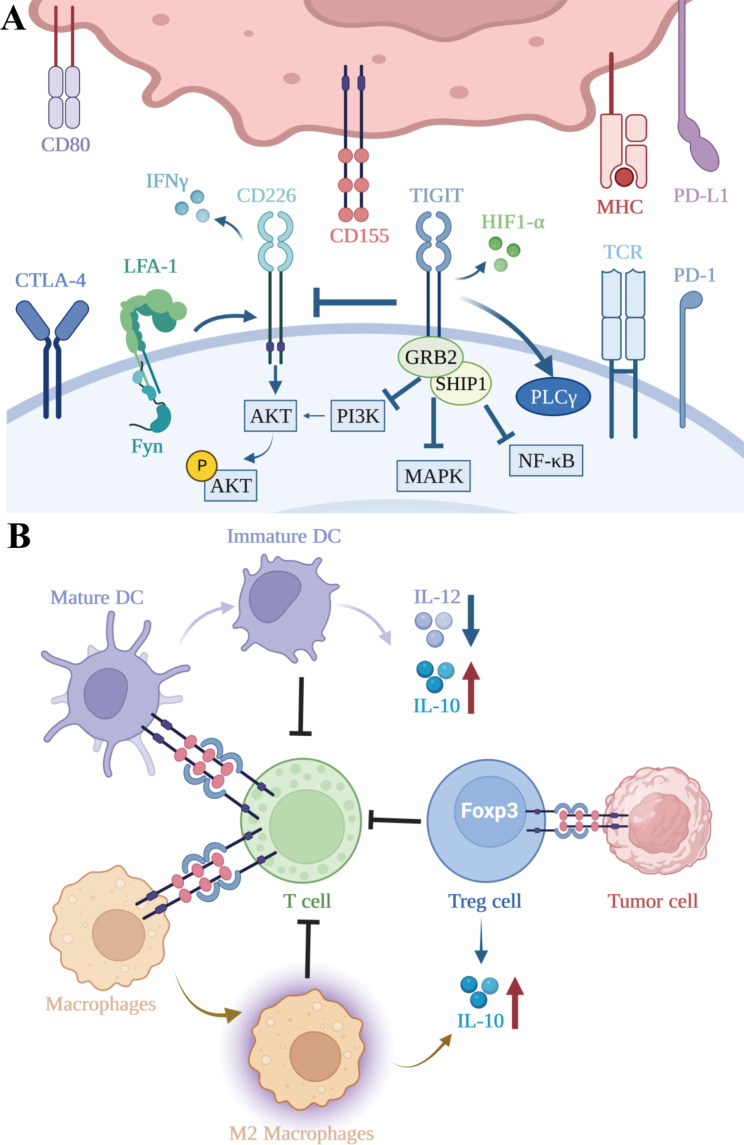
**(A)** Direct inhibitory effects of TIGIT. Firstly, TIGIT can directly inhibit the cytotoxic activity of T cells and NK cells by competitively antagonizing the stimulatory action of CD226. CD226 activation occurs upon binding with CD155 or CD112, which activates LFA-1, alters the conformation of ICAM-1, recruits Fyn, and drives the activation of the Akt signaling pathway, leading to the release of IFN-γ. Secondly, TIGIT can bind to CD155, and its ITT-like motif interacts with Grb2, which recruits SHIP1, thereby inhibiting PI3K, MAPK, and NF-κB signaling pathways. In addition, TIGIT also participates in the downregulation of the TCR complex itself and the central regulatory factors of TCR signaling cascades, such as PLCγ. TIGIT can also alter T cell metabolism by inhibiting glycolysis and synergizing with HIF1-α to enhance tumor cell invasion, colony formation, and angiogenesis. **(B)** Indirect inhibitory effects of TIGIT. Firstly, TIGIT exerts indirect inhibitory effects by triggering CD155 to induce DC acquisition of an immature tolerogenic phenotype, increasing IL-10 secretion, and decreasing IL-12 production. TIGIT can promote naive T cell differentiation into Treg cells more frequently and upregulate Foxp3 expression, which confers superior suppressive function to Treg cells. Finally, activation of the TIGIT/CD155 pathway can promote IL-10 transcription and induce macrophage polarization toward an anti-inflammatory M2 cytokine profile

The principal immunosuppressive mechanism of TIGIT is competing with CD226 to regulate T and NK cell functions, which is reminiscent of the B7-CD28-CTLA-4 pathway. On the one hand, TIGIT exerts its immunosuppressive effect by binding to CD155 and CD112 with a much higher affinity than that of CD226, thereby competitively inhibiting CD226 if both molecules are present on the same cell [21]. On the other hand, TIGIT directly interferes with the co-stimulatory function of CD226 by impeding its homodimerization [28]. By modulating CD226 activity, TIGIT can affect various T cell functions.CD226, also known as DNAX accessory molecule-1 (DNAM-1), was first identified by Shibuya as having a role in enhancing the cytotoxic function of T cells and NK cells [40]. CD226 mainly binds to and is activated by two cell surface ligands, CD155 and CD112, which are, like PD-1 ligands, typically over-expressed in tumors (Fig. 1A). Intracellularly, activated CD226 aggregates lymphocyte function-associated antigen 1 (LFA-1) to conformationally change intracellular adhesion molecule 1 (ICAM-1), which recruits Fyn and then drives activation of the Akt signaling pathway to promote NK/T cell-mediated tumor cytotoxicity [27, 41, 42]. In addition, CD226, by binding to CD155, triggers phosphorylation of FOXO1 [43], a transcription factor that negatively regulates homing and effector functions of NK cells [44]. Phosphorylated FOXO1 translocates from the nucleus to the cytoplasm for degradation by ubiquitination, which enables normal cell killing of NK cells [43]. Similarly, CD226-mediated inactivation of FOXO1 promotes T-cell survival, homing, proliferation, and differentiation [45]. Under the condition of IL-12-induced FOXO1 inactivation, CD8^+^ T cells acquire effector functions (KLRG1^hi^ phenotype) [44]. In addition, FOXO1 directly promotes *Eomes* transcription and differentiation into memory phenotypes of CD8^+^ T cells [45]. Moreover, CD226 assumes the role of an adhesion molecule that orchestrates the trans-endothelial migration of effector memory cells, enabling them to egress from circulation and infiltrate inflammatory foci, such as tumors [46]. CD226 also exerts a critical function in various stages of T cell activation by creating immune synapses with antigen-presenting cells (APCs) through interactions with CD155 [42]. Based on the data from CD226-deficient mice, Gilfillan concluded that CD226 plays an indispensable role in triggering the activation of CD8^+^ T cells in peripheral tissues, whereas it augments the ability of NK cells to execute cytotoxicity against tumor cells [47, 48]. Thus, by competing with CD226, TIGIT can inhibit the Akt signaling pathway and FOXO1 phosphorylation, suppress T/NK cell activation, migration, reduce cell toxicity, and promote T/NK cell exhaustion.

Lastly, overlay of genome-wide microarray data with T cell activation pathways showed that numerous molecules involved in T cell receptor (TCR) and CD28 signaling were significantly downregulated upon TIGIT binding [36]. The downregulation of TCRα chain, CD3ε, and PLCγ was confirmed via RT-PCR, suggesting that TIGIT induces downregulation of molecules that comprise the TCR complex and interferes with upstream of the TCR-induced signaling cascade [36]. While, other co-inhibitory molecules such as PD-1, interfere with processes further downstream in the signaling cascade [37]. Furthermore, TIGIT appears to have the capability of altering T cell metabolism via the blockade of glycolysis [38] and work in conjunction with hypoxia-inducible factor 1-α (HIF1-α) to increase tumor cell invasion, colony formation, and angiogenesis [39].

### Indirect inhibitory effects of TIGIT in tumor microenvironment

Dendritic cells (DCs) are sentinel antigen-presenting cells (APCs) that are responsible for capturing antigens, migrating, producing cytokines, and activating T cells and NK cells (Fig. 1B). However, it is only mature DCs that can activate T cells, while immature DCs can lead to unresponsiveness and/or tolerance to immunotherapy in T cells [49]. TIGIT could induce DCs to acquire an immature tolerogenic phenotype by triggering CD155, resulting in elevated IL-10 secretion and concomitant reduction in IL-12 production [25]. Since it prevents APCs from upregulating molecules involved in antigen presentation, IL-10 is critical for suppressing immune responses, thereby suppressing T cell proliferation and elaboration of immunostimulatory cytokines such as IFN-γ directly [50, 51].

Moreover, TIGIT is constitutively expressed on most Tregs and plays a vital role in their functioning and maintenance. First, TIGIT could promote naïve T cells to differentiate into Tregs more frequently and upregulate Foxp3 expression, which in turn confers superior suppressive function to Tregs [52]. Second, TIGIT^+^ Treg cells exhibit enhanced demethylation compared to their TIGIT^−^ Treg cell counterparts, resulting in higher lineage stability [53]. Third, TIGIT^+^ Treg cells express a highly immunosuppressive gene profile that restricts PI3K-AKT signaling, thereby inhibiting the acquisition of T helper 1 (Th1) and Th17 cell phenotypes [52]. In melanoma patients, Tregs that exhibit elevated levels of TIGIT expression are found to be enriched within tumor microenvironments and display a sustained immunosuppressive phenotype [54]. In a B16F10 melanoma model, transfer of TIGIT-deficient Tregs along with wild-type CD4^+^ and CD8^+^ T effector cells into tumor-bearing Rag mice has also been shown to markedly curtail tumor growth [55]. Hence, the therapeutic elimination of Tregs by means of anti-TIGIT antibody-dependent cytotoxicity may confer a considerable anti-tumor effect.

In addition to its effects on DCs and Tregs, activation of the TIGIT/CD155 pathway in macrophages could also increase IL-10 transcription and decrease IFN-γ through c-Maf nuclear translocation, while helping macrophages switch to anti-inflammatory M2 cytokine profiles [56]. In contradistinction, the introduction of TIGIT inhibitors could reprogram TIGIT^+^ M2 macrophages to the M1 phenotype, leading to increased CD47-mediated phagocytosis and ultimately benefiting the prognosis of patients with acute myeloid leukemia (AML) [57].

Moreover, MDSCs in the tumor microenvironment also play a critical role in curtailing anti-tumor immune responses. These cells exhibit heightened levels of CD155 and PD-L1, implying that their suppressive effects may be amplified via reverse signaling triggered by the TIGIT/CD155 and PD-1/PD-L1 pathways. Remarkably, anti-PD-L1 treatment augmented CD155 expression in MDSCs, whereas anti-TIGIT treatment upregulated PD-L1 expression [58].

### TIGIT in solid tumors and hematological malignancies

TIGIT is upregulated by T cells in a wide range of human solid tumors, such as lung cancer, urologic cancer, and breast cancer compared with normal tissue [28]. Taking into account the immunosuppressive properties of TIGIT, high-level TIGIT expression generally indicates poor prognosis in solid tumors. A meta-analysis showed that high expression of TIGIT indicated worse overall survival (OS) [hazard ratio (HR) 1.73; 95% confidence interval (95% CI) 1.50–1.99], progression-free survival (PFS) (HR 1.53, 95% CI 1.25–1.88), recurrence-free survival (HR 2.40, 95% CI 1.97–2.93), and disease-free survival (HR 6.57, 95% CI 0.73–59.16) in East Asian patients with solid tumors [59]. A study revealed high expression of CD155 in murine and human pancreatic adenocarcinoma cells and showed that the activation of the TIGIT/CD155 axis was critical in immune evasion [60]. Another study also showed that human gastric cancer cells interfered with CD8^+^ T-cell metabolism via the TIGIT/CD155 axis, impairing T-cell functionalities [61]. In patients with colorectal cancer, high TIGIT expression correlated with T cell exhaustion, advanced disease, early recurrence, and poor survival [62]. Contrarily, the study by Zhang et al. revealed that TIGIT inhibition prevented NK cell exhaustion and inhibited tumor growth in several tumor-bearing models, including those of colon cancer, breast cancer, and fibrosarcoma [63].

The expression of TIGIT is also typically upregulated and indicates poor clinical outcomes in several hematologic malignancies. First, in patients with chronic lymphocytic leukemia (CLL), AML, or adult acute lymphoblastic leukemia (ALL), TIGIT is commonly upregulated on CD4^+^ T cells, CD8^+^ T cells, Foxp3 + γδ T cells, or NK cells compared with healthy individuals [64–70]. Notably, TIGIT leads to CLL anergy by downregulating B cell receptor signaling [71]. It correlates with T cell exhaustion, NK cell dysfunction, unfavorable responses after chemotherapy, and leukemia relapse after allogeneic hematopoietic stem cell transplantation in AML patients [66, 67, 69, 70]. Similarly, high TIGIT expression results in lower secretion levels of IL-2, TNFα, and IFN-γ from T cells in ALL patients [64]. On the contrary, silencing TIGIT can restore normal functions of CD8^+^ T cells to release cytokines, such as TNFα, IFN-γ, IL-2, and IL-12, and decrease the susceptibility to apoptosis [64, 67]. Also, anti-TIGIT blockades can enhance NK cells’ cytotoxicity towards AML cells and repolarize M2 leukemia-associated macrophages into M1 phenotype and restore their phagocytic capabilities [57, 69]. Second, TIGIT also plays a critical role in patients with lymphoma. In a study, among TIGIT, lymphocyte-activation gene 3 protein (LAG-3), and CD96, only TIGIT was significantly increased after CAR-T cell therapy relapse in patients with mantle cell lymphoma (MCL) or other non-Hodgkin’s lymphomas, suggesting a central role of TIGIT in inhibiting normal T cell function in terms of MCL [72, 73]. Similarly, TIGIT expression was significantly higher in T cells from follicular lymphoma (FL) patients compared to healthy controls [74], and it correlated with dysfunctional TCR signaling and disease progression which can be restored by locking TIGIT [74, 75]. Third, TIGIT also has an impact on multiple myeloma (MM). TIGIT is upregulated on NK cells from MM patients and CD8^+^ T cells from mice or humans, playing a vital role in their exhaustion [76, 77]. Moreover, anti-TIGIT inhibitors could prevent T cell exhaustion [77], reduce tumor cell growth rate, prolong survival, and prevent myeloma escape after stem cell transplantation in mice with MM [78].

## Synergy of TIGIT blockades with PD-1/PD-L1 blockades

### Limitations of ICI monotherapy

PD-1, also known as CD279, is a transmembrane receptor expressed on activated immune cells, including T cells, NK cells, B cells, macrophages, DCs, and monocytes [79, 80]. Its cytoplasmic domains are involved in the formation of ITIMs and immunoreceptor tyrosine-based switch motifs (ITSMs), respectively [79, 81]. PD-1 interacts with two ligands, PD-L1 (also called B7-H1 or CD274) [82] and PD-L2 (also known as B7-H2 or CD273) [83]. PD-L1 is expressed on T cells, B cells, DCs, macrophages, and cancer cells, with high levels on cancer cell membranes [84]. The binding of cancer cell PD-L1 to PD-1 on T cells triggers negative signaling, inducing T cell apoptosis and impairing immunocompetence, thereby allowing cancer cells to evade immune surveillance and destruction [85]. Blocking the binding of PD-L1 to PD-1, which is the theoretical mechanism of PD-1/PD-L1 inhibitors, eliminates this negative feedback and restores the function of T cells, facilitating cancer cell killing [86].

Antibodies against the PD-1/PD-L1 pathway have been used in the treatment of several types of cancer, such as melanoma [87], lung cancer [88], lymphoma [89], and liver cancer [90]. However, despite the success in a small number of patients who experienced anti-cancer immunity recovery and long-term remission, the response rate of PD-1/PD-L1 blockades is low in general [91, 92]. This treatment is also limited by the lack of effective biomarkers [93], immune-related toxicity [94], and innate and acquired drug resistance [95, 96]. Numerous mechanisms contribute to resistance to anti-PD-1/PD-L1 therapy, such as T cell exclusion and exhaustion, local immune dysfunction, loss of neoantigens or PD-L1, signaling defects, as well as non-immune factors including metabolism, epigenetics, and microbiota [97]. Upregulations of coinhibitory molecules, such as TIGIT, LAG-3, and V domain immunoglobulin suppressor of T cell activation (VISTA), account for a significant factor for T cell dysfunction and subsequent resistance to anti-PD-1/PD-L1 therapies for a number of patients [97]. TIGIT inhibition not only enhances CD8 T-cell cytotoxicity but also boosts NK cell anti-tumor responses. Consequently, blocking TIGIT is promising immunotherapy. However, Vibostolimab and Tiragolumab monotherapies show null objective response rates (ORR) [98, 99]. Fortunately, Tiragolumab combined with Atezolizumab achieves 37% ORR overall and 66% in PD-L1 TPS > 50% subset, surpassing Atezolizumab monotherapy (21% and 24% response rates, respectively) [24].

Based on these findings, the use of dual checkpoint inhibition could potentially enhance the restoration of anti-tumor immunity and lead to improved efficacy of immunotherapy for a wider range of cancer patients.

### Molecular basis of TIGIT and PD-1/PD-L1 co-inhibition

TIGIT is typically co-expressed with PD-1 on a wide variety of T cells. Moreover, PD-1 blockade could increase TIGIT expression on CD8^+^ T cells by 1.5 folds [22]. Using a gene signature-based approach, Johnston et al. investigated the gene expression data in lung cancer and found a strong correlation between TIGIT expression and the infiltration of CD8^+^ T cells, as well as the expression of PD-1 on these cells [28]. Among the inhibitor receptors that are co-expressed with TIGIT, PD-1 is preferentially co-expressed [100]. And TIGIT is also the most frequent co-expressed immune checkpoint receptor on PD-1^+^ CD8^+^ T cells [101]. Furthermore, the co-expression of TIGIT and PD-1 manifested immuno-suppressive phenotypes of exhausted T cells or Tregs [22]. Based on these observations, the monitoring of co-expression of TIGIT and PD-1 was proposed as a predictive biomarker for the clinical efficacy of ICIs in various cancers [102, 103].

**Fig. 2:**
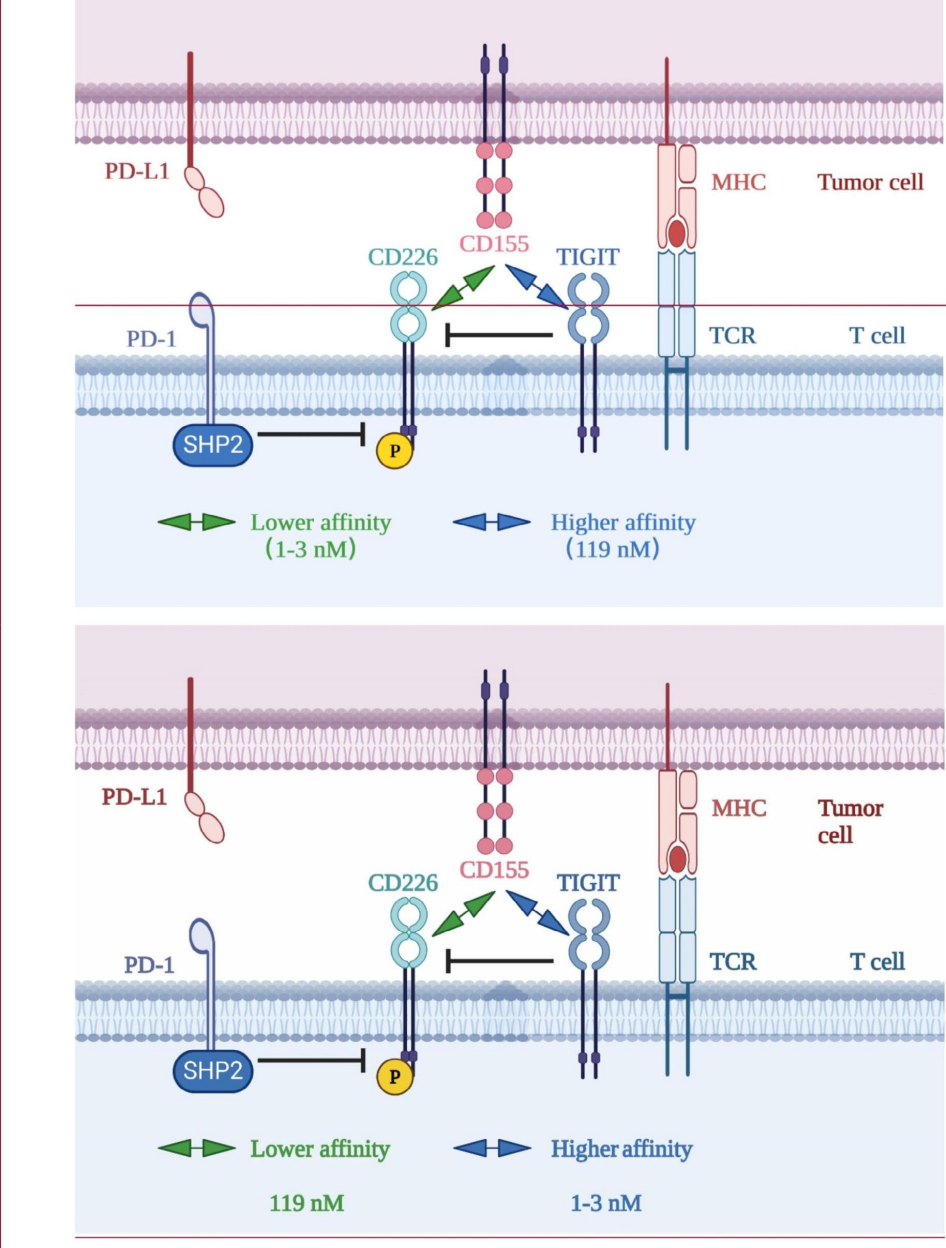
Mechanism of co-inhibition by TIGIT and PD-1. The TIGIT/CD226 pathway and the PD-1/PD-L1 pathway have an intersecting crossroad. On the one hand, upon activation by PD-L1, the intracellular domain of PD-1 recruits Shp2 to dephosphorylate CD226, inhibiting the immune activation function of CD226. On the other hand, TIGIT has a higher affinity (dissociation constant 1–3 nM) to CD155 than that of CD226 (dissociation constant 119 nM) [25], thus competitively antagonizes and blocks CD226 homodimerization through its extracellular domain, inhibiting the immune activation function of CD226.

Banta’s study demonstrated that CD226 expression is necessary for the effectiveness of PD-(L)1 or TIGIT co-blockades [23]. Firstly, PD-1 and TIGIT can independently inhibit CD226 functionality. The mechanistic investigations further revealed that TIGIT inhibited CD226 by competitive binding to the shared ligands through its extracellular domain, while the intracellular domain of PD-1, following activation of PD-1, recruits Shp2 to dephosphorylate CD226 (Fig. 2) [23, 104]. Secondly, the study revealed that anti-PD-1 treatment appears to be more efficient than anti-TIGIT therapy in yielding CD226 activation. The ligand competition effect is less apparent when the ligands are overexpressed. Specifically, if CD155, a ligand of both TIGIT and CD226, is overexpressed, CD226 would also become activated extracellularly. However, the intracellular activation of CD226 is dependent on Shp2 without the involvement of CD155. Thirdly, when both PD-1 and TIGIT are co-expressed, far less phosphorylated CD226 is detected than when either is expressed alone. In other words, the presence of TIGIT prevents a stand-alone PD-1/PD-L1 inhibitor from fully activating CD226, demonstrating that only combining anti-TIGIT with PD-1/PD-L1 blockade may fully activate CD226 [23].

Further investigations suggest that dual blockade of TIGIT and PD-1 has the potential to serve as an effective anticancer therapy. Thibaudin et al. assessed the potential of combining atezolizumab (anti-PD-L1) and tiragolumab (anti-TIGIT) to reinvigorate the immune response of tumor-infiltrating lymphocytes in microsatellite-stable (MSS) colorectal cancer [106]. While atezolizumab alone only reinvigorates T cells in microsatellite-unstable tumors, the combined use of atezolizumab and tiragolumab can reinvigorate T cells in 46% of MSS colorectal cancer samples [106]. Hung’s study, using a mouse model with intracranial GL261-luc tumors, showed a significant improvement in survival rate using dual therapy with anti-PD-1 and anti-TIGIT compared with control and single-agent groups [107]. Hansen evaluated the combined use of COM902, an anti-TIGIT antibody, and a PD-L1 inhibitor in CT26 colon cancer and renca renal cancer models and found that the combination therapy significantly improved overall survival compared to PD-L1 inhibitor monotherapy [108]. Besides, dual blockade of the TIGIT and PD-1/PD-L1 pathways has yielded favorable prognoses in various animal models, including the SGC7901 [61], MC38-CEA, TC1 [109], pancreatic cancer [105], and cervical cancer models [110].

In addition to solid tumors, similar investigations have been conducted on hematological malignancies [111]. Wang et al. conducted a study on patients with AML and found increased PD-1 and TIGIT expression as well as decreased CD226 expression in peripheral blood CD8^+^ T cells compared with those of healthy individuals, and these cells were crucial biomarkers of poor clinical prognosis [112]. Furthermore, high PD-1 and TIGIT expression are closely associated with late leukemia relapse after CAR-T therapy [113]. Zhang’s research revealed that a single TIGIT inhibitor upregulated only IFN-γ and TNF-α, but the combination of anti-TIGIT and anti-PD-1 inhibitors significantly upregulated IL-2, IFN-γ, and TNF-α in CD4^+^ or CD8^+^ T cells, which could enhance anti-leukemia immune response [64]. Among four different checkpoint combinations, PD-1/TIM-3, PD-1/LAG-3, PD-1/CTLA-4, and PD-1/TIGIT, Lee et al. discovered that CAR-T cells with downregulated PD-1 and TIGIT displayed strong anti-tumor activity and significantly improved the prognosis of diffuse large B-cell lymphoma patients [114]. Functional and phenotypic analysis showed that downregulation of PD-1 enhanced short-term effector function, while downregulation of TIGIT mainly led to a less exhausted cell state [113]. In conclusion, these studies support the co-inhibition of TIGIT and PD-1/PD-L1 in treating hematological malignancies.

## Clinical studies on TIGIT and PD-1/PD-L1 co-inhibition

Currently, a variety of novel drugs or combination strategies targeting the co-inhibition of TIGIT and PD-1/PD-L1 are under evaluation in clinical trials. A summary of these clinical trials registered on clinicaltrials.gov is provided in Table 1. Terminated or withdrawn clinical trials resulting from various factors are excluded from consideration. Generally, there are three types of these treatments in Table 2: (1) simultaneous administrations of anti-TIGIT and anti-PD-1/PD-L1 agents (for example, tiragolumab plus atezolizumab); (2) coformulation of anti-TIGIT and anti-PD-1/PD-L1 agents (e.g., MK-7684 A, which is a coformulation of pembrolizumab and vibostolimab); (3) bispecific antibodies binding both TIGIT and PD-1/PD-L1 (such as IBI321). The evaluations are taken on a wide range of solid tumors and hematological malignancies at various lines and distinct situations, such as neoadjuvant, adjuvant, and palliative treatments (Table 1). Some of the studies aim further to assess the combination of anti-TIGIT and anti-PD-1/PD-L1 treatments with other therapies, including chemotherapy, radiation therapy, concurrent chemoradiotherapy, and targeted therapies.

**Table 1:** Clinical trials on the bi-inhibition of TIGIT and PD-1/PD-L1 registered on clinicaltrials.gov

No	NCT number			Phase		Status		Cancer		Drug(s) of Intervention Arm(s)		Drug(s) of Control Arm(s)		Outcomes
1	NCT04948697			2		Active, not recruiting		Advanced Liver Cancers		Ociperlimab plus Tislelizumab plus BAT1706		Tislelizumab plus BAT1706		-
2	NCT05724563			2		Not yet recruiting		Advanced Liver Cancers		Zimberelimab plus Domvanalimab		-		-
3	NCT04524871			1/2		Recruiting		Advanced Liver Cancers		Atezolizumab plus Bevacizumab plus Tiragolumab		Atezolizumab plus Bevacizumab Atezolizumab plus Bevacizumab plus Tocilizumab Atezolizumab plus Bevacizumab plus TPST-1120 RO7247669 plus Bevacizumab Atezolizumab plus Bevacizumab plus ADG126		-
4	NCT04911894			1		Completed		Advanced Solid Tumor		IBI321		-		-
5	NCT04911881			1		Completed		Advanced Solid Tumor		IBI321		-		-
6	NCT02913313			1/2		Active, not recruiting		Advanced Solid Tumor		BMS-986,207 plus nivolumab BMS-986,207 plus nivolumab plus ipilimumab BMS-986,207		-		-
7	NCT02794571			2		Active, not recruiting		Advanced Solid Tumor		Atezolizumab plus Tiragolumab		-		ORR_dual_: 46% (6/13) DCR_dual_: 85% (11/13)
8	NCT02964013			1		Active, not recruiting		Advanced Solid Tumor		Vibostolimab plus pembrolizumab Vibostolimab plus pembrolizumab plus chemotherapy MK-7684 A Vibostolimab plus chemotherapy Vibostolimab		-		ORR_dual_: 26% mPFS_dual_: 8.4 months
9	NCT04570839			1/2		Active, not recruiting		Advanced Solid Tumor		COM701 plus BMS-986,207 plus Nivolumab		-		ORR_dual_: 20% (4/20) DCR_dual_: 45% (9/20)
10	NCT03628677			1		Active, not recruiting		Advanced Solid Tumor		Domvanalimab plus Zimberelimab Domvanalimab		-		-
11	NCT04457778			1		Active, not recruiting		Advanced Solid Tumor		M6223 plus Bintrafusp alfa M6223		-		-
12	NCT04632992			2		Active, not recruiting		Advanced Solid Tumor		Atezolizumab plus Tiragolumab		Entrectinib Inavolisib Alectinib Ipatasertib Atezolizumab plus Investigator’s Choice of Chemotherapy Trastuzumab Emtansine plus Atezolizumab Pertuzumab plus Trastuzumab plus Hyaluronidase-zzxf Pertuzumab plus Trastuzumab plus Hyaluronidase-zzxf plus Investigator’s Choice of Chemotherapy Trastuzumab Emtansine plus Tucatinib Trastuzumab Emtansine plus Atezolizumab Ipatasertib plus Atezolizumab Ipatasertib plus Paclitaxel Pralsetinib		-
13	NCT03260322			1		Completed		Advanced Solid Tumor		ASP8374 plus Pembrolizumab ASP8374		-		-
14	NCT04353830			1		Completed		Advanced Solid Tumor		IBI939 plus Sintilimab IBI939		-		-
15	NCT04335253			1		Completed		Advanced Solid Tumor		EOS-448		-		-
16	NCT03945253			1		Completed		Advanced Solid Tumor		ASP8374		-		-
17	NCT05172856			1		Not yet recruiting		Advanced Solid Tumor		IBI321		-		-
18	NCT05483400			2		Not yet recruiting		Advanced Solid Tumor		Tiragolumab plus Atezolizumab		-		-
19	NCT05259319			1		Not yet recruiting		Advanced Solid Tumor		Atezolizumab plus Tiragolumab plus SBRT		-		-
20	NCT05537051			1		Not yet recruiting		Advanced Solid Tumor		PM1021 plus PM8001 PM1021		-		-
21	NCT05715281			2		Not yet recruiting		Advanced Solid Tumor		Atezolizumab plus Tiragolumab		-		-
22	NCT05661578			2		Recruiting		Advanced Solid Tumor		Tiragolumab plus Atezolizumab		-		-
23	NCT05286801			1/2		Recruiting		Advanced Solid Tumor		Atezolizumab plus Tiragolumab		-		-
24	NCT03977467			2		Recruiting		Advanced Solid Tumor		Atezolizumab plus Tiragolumab		Atezolizumab plus chemotherapy		-
25	NCT04047862			1		Recruiting		Advanced Solid Tumor		Ociperlimab plus Tislelizumab Ociperlimab plus Tislelizumab plus chemotherapy Ociperlimab		-		ORR_dual_: 57.5% vs. ORR_dual+C_: 54.8%
26	NCT05417321			1/2		Recruiting		Advanced Solid Tumor		HB0036		-		-
27	NCT05026606			2		Recruiting		Advanced Solid Tumor		Etigilimab plus Nivolumab		-		-
28	NCT04761198			1/2		Recruiting		Advanced Solid Tumor		Etigilimab plus Nivolumab		-		-
29	NCT04354246			1		Recruiting		Advanced Solid Tumor		COM902 plus COM701 plus Pembrolizumab COM902 plus COM701 COM902		-		-
30	NCT05120375			1		Recruiting		Advanced Solid Tumor		BAT6021		-		-
31	NCT05073484			1		Recruiting		Advanced Solid Tumor		BAT6021 plus BAT1308 BAT6021		-		-
32	NCT05060432			1/2		Recruiting		Advanced Solid Tumor		EOS-448 plus Pembrolizumab EOS-448 plus Inupadenant EOS-448 plus Dostarlimab EOS-448 plus Dostarlimab plus Inupadenant EOS-448 plus Dostarlimab plus chemotherapy		Inupadenant plus Dostarlimab		-
33	NCT05007106			2		Recruiting		Advanced Solid Tumor		MK-7684 A MK-7684 A plus Lenvatinib MK-7684 A plus 5-Fluorouracil plus Cisplatin MK-7684 A plus Paclitaxel MK-7684 A plus Gemcitabine/Cisplatin MK-7684 A plus Carboplatin/Paclitaxel/Bevacizumab MK-7684 A plus Capecitabine/Oxaliplatin		Pembrolizumab		-
34	NCT04446351			1		Recruiting		Advanced Solid Tumor		EOS-448 plus Dostarlimab EOS-448 plus Dostarlimab plus GSK6097608		GSK6097608 GSK6097608 plus Dostarlimab Dostarlimab Dostarlimab plus Cobolimab		-
35	NCT03869190			1/2		Recruiting		Advanced Solid Tumor		Atezolizumab plus Tiragolumab		Atezolizumab Atezolizumab plus Enfortumab Vedotin Atezolizumab plus Niraparib Atezolizumab plus Magrolimab Atezolizumab plus Sacituzumab Govitecan Atezolizumab plus RO7122290 Cisplatin		-
36	NCT05757492			1/2		Recruiting		Advanced Solid Tumor		JS006 plus Toripalimab		-		-
37	NCT05394337			1		Not yet recruiting		Advanced Urothelial Carcinoma		Neoadjuvant atezolizumab plus Tiragolumab		-		-
38	NCT05845814			1/2		Not yet recruiting		Advanced Urothelial Carcinoma		MK-7684 A plus EV		Coformulated Favezelimab/Pembrolizumab plus EV Pembrolizumab plus EV		-
39	NCT05327530			2		Recruiting		Advanced Urothelial Carcinoma		Avelumab plus M6223		Avelumab Avelumab plus Sacituzumab Govitecan Avelumab plus NKTR-255		-
40	NCT03547973			2		Recruiting		Advanced Urothelial Carcinoma		Sacituzumab Govitecan-hziy plus Zimberelimab plus Domvanalimab		Sacituzumab Govitecan-hziy Sacituzumab Govitecan-hziy plus Pembrolizumab Sacituzumab Govitecan-hziy plus Cisplatin plus Avelumab Sacituzumab Govitecan-hziy plus Cisplatin plus Zimberelimab Sacituzumab Govitecan-hziy plus Zimberelimab Avelumab Zimberelimab Carboplatin plus Gemcitabine		-
41	NCT05645692			2		Recruiting		Advanced Urothelial Carcinoma		Tiragolumab plus R07247669		RO7247669 Atezolizumab		-
42	NCT05023109			2		Recruiting		Biliary Tract Carcinoma		Tislelizumab plus Ociperlimab plus chemotherapy		-		-
43	NCT02625961			2		Recruiting		Bladder Cancer		MK-7684 A		Pembrolizumab		-
44	NCT04693234			2		Active, not recruiting		Cervical Cancer		Tislelizumab plus Ociperlimab		Tislelizumab		-
45	NCT04300647			2		Active, not recruiting		Cervical Cancer		Tiragolumab plus Atezolizumab		Atezolizumab		-
46	NCT04895722			2		Recruiting		Colorectal Cancer		MK-7684 A		Pembrolizumab Co-formulated Pembrolizumab/Quavonlimab Co-formulated Pembrolizumab/Favezelimab Pembrolizumab Plus MK-4830		-
47	NCT04929223			1		Recruiting		Colorectal Cancer		Atezolizumab plus Tiragolumab plus Bevacizumab Atezolizumab plus Tiragolumab		Inavolisib plus Cetuximab Inavolisib plus Bevacizumab Atezolizumab plus SY-5609 GDC-6036 plus Cetuximab plus FOLFOX GDC-6036 plus Cetuximab		-
48	NCT04486352			1/2		Recruiting		Endometrial Cancer		Atezolizumab plus Tiragolumab		Atezolizumab plus Bevacizumab Atezolizumab plus Ipatasertib Atezolizumab plus Talazoparib Atezolizumab plus Trastuzumab emtansine (TDM-1) Inavolisib plus Letrozole		-
49	NCT04540211			3		Active, not recruiting		ESCC		Tiragolumab plus Atezolizumab plus chemotherapy		Placebo plus chemotherapy		-
50	NCT05743504			1/2		Not yet recruiting		ESCC		Tiragolumab plus Atezolizumab with CCRT before surgery		-		-
51	NCT04732494			2		Recruiting		ESCC		Tislelizumab plus Ociperlimab		Tislelizumab plus Placebo		-
52	NCT04543617			3		Recruiting		ESCC		Tiragolumab plus Atezolizumab Tiragolumab		Atezolizumab plus Placebo		-
53	NCT03281369			1/2		Recruiting		ESCC		Atezolizumab plus Tiragolumab plus chemotherapy Atezolizumab plus Tiragolumab		Atezolizumab		-
54	NCT04933227			2		Active, not recruiting		Gastric Cancer		Tiragolumab plus Atezolizumab plus chemotherapy		-		-
55	NCT05251948			1/2		Active, not recruiting		Gastric Cancer		Tiragolumab plus Atezolizumab plus chemotherapy		Atezolizumab plus chemotherapy		-
56	NCT05568095			3		Recruiting		Gastric Cancer		Zimberelimab plus Domvanalimab plus chemotherapy		Nivolumab plus chemotherapy		-
57	NCT05329766			2		Recruiting		Gastric Cancer		Domvanalimab plus Zimberelimab plus chemotherapy Domvanalimab plus Zimberelimab		Zimberelimab plus chemotherapy Zimberelimab plus Quemliclustat		-
58	NCT05702229			2		Recruiting		Gastric Cancer		AZD2936 Plus XELOX/FOLFOX		MEDI5752 plus XELOX/FOLFOX		-
59	NCT04826393			1		Active, not recruiting		Glioblastoma		ASP8374 plus Cemiplimab		-		-
60	NCT04656535			1		Recruiting		Glioblastoma		AB122 plus Domvanalimab plus surgery AB122 plus Domvanalimab Domvanalimab plus surgery		AB122 plus surgery surgery		-
61	NCT05130177			2		Recruiting		Melanoma		Zimberelimab plus Domvanalimab		-		-
62	NCT05665595			3		Recruiting		Melanoma		Pembrolizumab plus Vibostolimab		Pembrolizumab		-
63	NCT05060003			2		Recruiting		Melanoma		Atezolizumab plus Tiragolumab		Atezolizumab		-
64	NCT04305041			1/2		Recruiting		Melanoma		Pembrolizumab plus Quavonlimab plus Vibostolimab		Pembrolizumab plus Quavonlimab plus Lenvatinib Pembrolizumab plus all-trans retinoic acid (ATRA)		-
65	NCT04305054			1/2		Recruiting		Melanoma		Pembrolizumab plus Vibostolimab Coformulation Favezelimab/Pembrolizumab plus Vibostolimab		Pembrolizumab Coformulation Pembrolizumab/Quavonlimab Coformulation Pembrolizumab/Quavonlimab plus Lenvatinib Coformulation Favezelimab/Pembrolizumab Coformulation Favezelimab/Pembrolizumab plus All-trans Retinoic Acid (ATRA)		-
66	NCT04303169			1/2		Recruiting		Melanoma		Pembrolizumab plus Vibostolimab		Pembrolizumab plus Gebasaxturev Pembrolizumab Pembrolizumab plus MK-4830 Favezelimab plus Pembrolizumab Pembrolizumab plus all-trans retinoic acid (ATRA)		pCR_dual_: 38% vs. pCR_P_: 40% pPR_dual_: 31% vs. pPR_P_: 27% RFS_dual_ (18 m): 95% vs. RFS_P_ (18 m): 73% ORR_dual_: 50% vs. ORR_P_: 27% EFS_dual_ (18 m): 81% vs. EFS_P_ (18 m): 79%
67	NCT03554083			2		Recruiting		Melanoma		Atezolizumab plus Tiragolumab		Vemurafenib plus Cobimetinib plus Atezolizumab Cobimetinib plus Atezolizumab		-
68	NCT05116202			1/2		Recruiting		Melanoma		Atezolizumab plus Tiragolumab RO7247669 plus Tiragolumab		Nivolumab plus Ipilimumab RO7247669		-
69	NCT02861573			1/2		Recruiting		Metastatic Castration-Resistant Prostate Cancer		MK-7684 A		Pembrolizumab plus Olaparib Pembrolizumab plus Docetaxel plus Prednisone Pembrolizumab plus Enzalutamide Pembrolizumab plus Abiraterone plus Prednisone Pembrolizumab plus Lenvatinib Pembrolizumab plus Carboplatin plus Etoposide Carboplatin plus Etoposide Belzutifan Pembrolizumab plus Belzutifan		-
70	NCT04672356			1		Active, not recruiting		NSCLC		IBI939 plus Sintilimab		-		-
71	NCT04672369			1		Active, not recruiting		NSCLC		IBI939 plus Sintilimab		Sintilimab		ORR_dual_: 66.7% (18/27) vs. ORR_P_: 61.5% (8/13) mPFS_dual_: NR vs. mPFS_P_: 6.0 m (HR = 0.43)
72	NCT03563716			2		Active, not recruiting		NSCLC		Atezolizumab plus Tiragolumab		Atezolizumab plus Placebo		ORRdual: 31.3% vs. ORRP: 16.2% mPFSdual: 5.4 m vs. mPFSP: 3.6 m (HR = 0.57)
73	NCT04725188			2		Active, not recruiting		NSCLC		MK-7684 A plus chemotherapy MK-7684 A		chemotherapy		Did not reach statistical significance for PFS and was numerically less effective than Docetaxel
74	NCT04262856			2		Active, not recruiting		NSCLC		Zimberelimab plus Domvanalimab plus Etrumadenant Zimberelimab plus Domvanalimab		Zimberelimab		ORR_dual+O_: 40% vs. ORR_dual_: 41% vs. ORR_P_: 27% mPFS_dual+O_: 10.9 m vs. mPFS_P_: 5.4 m (HR = 0.65) mPFS_dual_: 12.0 m vs. mPFS_P_: 5.4 m (HR = 0.55)
75	NCT03819465			1		Active, not recruiting		NSCLC		AZD2936 AZD2936 plus chemotherapy		Durvalumab Durvalumab plus danvatirsen Durvalumab plus oleclumab MEDI5752 Durvalumab plus Investigator’s choice of chemotherapy Durvalumab plus Investigator’s choice of chemotherapy plus danvatirsen Durvalumab plus investigator’s choice of chemotherapy plus oleclumab		-
76	NCT04585815			2		Active, not recruiting		NSCLC		SEA-TGT plus sasanlimab plus Axitinib		Sasanlimab plus Encorafenib plus inimetinib		-
77	NCT05034055			2		Not yet recruiting		NSCLC		Atezolizumab plus Tiragolumab plus SBRT		-		-
78	NCT05798663			2		Not yet recruiting		NSCLC		Atezolizumab plus Tiragolumab		Atezolizumab		-
79	NCT05825625			2		Not yet recruiting		NSCLC		Tiragolumab plus Atezolizumab with chemotherapy		-		-
80	NCT05746481			2		Not yet recruiting		NSCLC		Tiragolumab plus Atezolizumab plus Pemetrexed plus Carboplatin		-		-
81	NCT05791097			3		Not yet recruiting		NSCLC		Ociperlimab + Tislelizumab + chemotherapy		Placebo + Pembrolizumab + chemotherapy Placebo + Tislelizumab + chemotherapy		-
82	NCT04995523			1/2		Recruiting		NSCLC		AZD2936		-		-
83	NCT04791839			2		Recruiting		NSCLC		Zimberelimab plus Domvanalimab plus Etrumadenant		-		-
84	NCT05211895			3		Recruiting		NSCLC		Durvalumab plus Domvanalimab		Durvalumab plus Placebo		-
85	NCT04746924			3		Recruiting		NSCLC		Tislelizumab plus Ociperlimab		Pembrolizumab plus Placebo Tislelizumab plus Placebo		-
86	NCT04294810			3		Recruiting		NSCLC		Atezolizumab plus Tiragolumab		Atezolizumab plus Placebo		-
87		NCT04958811	2		Recruiting		NSCLC		Tiragolumab plus Atezolizumab plus Bevacizumab		-		-	
88		NCT04513925	3		Recruiting		NSCLC		Atezolizumab plus Tiragolumab		Durvalumab		-	
89		NCT05014815	2		Recruiting		NSCLC		Ociperlimab plus Tislelizumab plus chemotherapy		Placebo plus Tislelizumab plus chemotherapy		-	
90		NCT04619797	2/3		Recruiting		NSCLC		Tiragolumab plus Atezolizumab plus chemotherapy		Placebo plus Pembrolizumab plus chemotherapy		-	
91		NCT05502237	3		Recruiting		NSCLC		Zimberelimab plus Domvanalimab plus chemotherapy		Pembrolizumab plus chemotherapy Zimberelimab plus chemotherapy		-	
92		NCT04738487	3		Recruiting		NSCLC		MK-7684 A (Pembrolizumab/Vibostolimab)		Pembrolizumab		-	
93		NCT05298423	3		Recruiting		NSCLC		MK-7684 A (Pembrolizumab/Vibostolimab) plus chemotherapy plus radiotherapy		Durvalumab plus chemotherapy plus radiotherapy		-	
94		NCT04832854	2		Recruiting		NSCLC		Tiragolumab plus Atezolizumab Tiragolumab plus Atezolizumab plus chemotherapy		chemotherapy		-	
95		NCT05226598	3		Recruiting		NSCLC		MK-7684 A plus chemotherapy		Pembrolizumab plus chemotherapy		-	
96		NCT04866017	3		Recruiting		NSCLC		Ociperlimab plus Tislelizumab plus chemoradiotherapy		Tislelizumab plus chemoradiotherapy Durvalumab plus chemoradiotherapy		-	
97		NCT05102214	1/2		Recruiting		NSCLC		HLX301		-		-	
98		NCT04736173	3		Recruiting		NSCLC		Zimberelimab plus Domvanalimab		Carboplatin plus paclitaxel or pemetrexed Zimberelimab		-	
99		NCT04612751	1		Recruiting		NSCLC		Datopotamab deruxtecan plus AZD2936 Datopotamab deruxtecan plus AZD2936 plus Carboplatin		Datopotamab deruxtecan plus Durvalumab Datopotamab deruxtecan plus Durvalumab plus Carboplatin Datopotamab deruxtecan plus MEDI5752 Datopotamab deruxtecan plus MEDI5752 plus Carboplatin		-	
100		NCT05676931	2		Recruiting		NSCLC		Domvanalimab plus Zimberelimab Domvanalimab plus Zimberelimab plus Platinum Doublet Chemotherapy Domvanalimab plus Quemliclustat plus Zimberelimab plus Platinum Doublet Chemotherapy Domvanalimab plus Zimberelimab plus Docetaxel		Quemliclustat plus Zimberelimab Quemliclustat plus Zimberelimab plus Platinum Doublet Chemotherapy Quemliclustat plus Zimberelimab plus Docetaxel		-	
101		NCT05633667	2		Recruiting		NSCLC		Sacituzumab Govitecan-hziy plus Zimberelimab plus Domvanalimab Etrumadenant plus Zimberelimab plus Domvanalimab		Etrumadenant plus Zimberelimab Zimberelimab plus Platinum Based Chemotherapy Etrumadenant plus Zimberelimab plus Sacituzumab Govitecan-hziy Either Docetaxel or Sacituzumab Govitecan-hziy monotherapy		-	
102		NCT05565378	2		Recruiting		NSCLC		EOS-448 plus Dostarlimab		Pembrolizumab Monotherapy Dostarlimab Monotherapy		-	
103		NCT03739710	2		Recruiting		NSCLC		EOS-448 plus Dostarlimab EOS-448 plus Dostarlimab plus GSK6097608		feladilimab plus ipilimumab Docetaxel Docetaxel plus Feladilimab		-	
104		NCT04165070	2		Recruiting		NSCLC		Pembrolizumab plus Vibostolimab plus Carboplatin plus Paclitaxel Pembrolizumab plus Vibostolimab plus Carboplatin plus Pemetrexed		Pembrolizumab plus Boserolimab plus Carboplatin plus Paclitaxel Pembrolizumab plus Boserolimab plus Carboplatin plus Pemetrexed Pembrolizumab plus MK-4830 plus Carboplatin plus Paclitaxel Pembrolizumab plus MK-4830 plus Carboplatin plus Pemetrexed Pembrolizumab plus MK-0482 plus Carboplatin plus Paclitaxel Pembrolizumab plus MK-0482 plus Carboplatin plus Pemetrexed		-	
105		NCT05577702	2		Recruiting		NSCLC		Tislelizumab and Ociperlimab		Tislelizumab Monotherapy Tislelizumab and LBL-007		-	
106		NCT05681039	2		Not yet recruiting		Oral Cavity Squamous Cell Carcinoma		Tiragolumab Plus Atezolizumab		-		-	
107		NCT05715216	2		Recruiting		Ovarian Cancer		Etigilimab plus Nivolumab		-		-	
108		NCT05419479	1/2		Recruiting		Pancreatic Cancer		domvanalimab plus Zimberelimab plus APX005M		FOLFIRI		-	
109		NCT03193190	1/2		Recruiting		Pancreatic Cancer		Atezolizumab plus Chemotherapy plus Tiragolumab		Nab-Paclitaxel and Gemcitabine Atezolizumab plus Chemotherapy plus Selicrelumab Atezolizumab plus Chemotherapy plus Bevacizumab Atezolizumab plus Chemotherapy plus AB928 Atezolizumab plus Cobimetinib Atezolizumab plus PEGPH20 Atezolizumab plus BL-8040 Atezolizumab plus RO6874281 Nab-Paclitaxel and Gemcitabine or mFOLFOX6 Atezolizumab plus Chemotherapy plus Tocilizumab		-	
110		NCT05009069	2		Recruiting		Rectal Cancer		Tiragolumab plus Atezolizumab plus radiotherapy		Atezolizumab		-	
111		NCT04626479	1/2		Recruiting		Renal Cell Carcinoma		MK-7684 A plus Belzutifan		Coformulation Pembrolizumab/Quavonlimab plus Lenvatinib Coformulation Favezelimab/Pembrolizumabplus Lenvatinib Pembrolizumab plus Belzutifan plus Lenvatinib Pembrolizumab plus Lenvatinib		-	
112		NCT05805501	2		Recruiting		Renal Cell Carcinoma		RO7247669 plus Tiragolumab plus Axitinib		RO7247669 plus Axitinib Pembrolizumab plus Axitinib		-	
113		NCT04952597	2		Active, not recruiting		SCLC		Ociperlimab plus Tislelizumab plus chemoradiotherapy		Tislelizumab plus Concurrent Chemoradiotherapy Concurrent Chemoradiotherapy		-	
114		NCT04256421	3		Active, not recruiting		SCLC		Tiragolumab plus Atezolizumab plus chemotherapy		Placebo plus Atezolizumab plus chemotherapy		mPFS_dual+C_: 5.4 m vs. mPFS_P+C_: 5.6 m (HR = 1.11) mOS_dual+C_: 13.6 m vs. mOS_P+C_: 13.6 m (HR = 1.04) DoR_dual+C_: 4.2 m vs. DoR_P+C_: 5.1 m	
115		NCT04308785	2		Active, not recruiting		SCLC		Atezolizumab plus Tiragolumab		Atezolizumab plus Placebo		-	
116		NCT04665856	3		Active, not recruiting		SCLC		Tiragolumab plus Atezolizumab plus chemotherapy		Placebo plus Atezolizumab plus chemotherapy		-	
117		NCT05224141	3		Recruiting		SCLC		MK-7684 A plus chemotherapy		Atezolizumab plus chemotherapy		-	
118		NCT04665843	2		Active, not recruiting		Squamous Cell Carcinoma of Head and Neck		Atezolizumab plus Tiragolumab		Atezolizumab plus Placebo		-	
119		NCT03708224	2		Recruiting		Squamous Cell Carcinoma of Head and Neck		Atezolizumab plus Tiragolumab		Atezolizumab plus Tocilizumab Atezolizumab Monotherapy Atezolizumab (Adjuvant)		-	
120		NCT05459129	1/2		Recruiting		Squamous Cell Carcinoma of Head and Neck		Atezolizumab plus Tiragolumab Atezolizumab plus Tiragolumab plus Carboplatin plus Paclitaxel		-		-	
121		NCT05661188	2		Not yet recruiting		Squamous Cell Carcinoma of the Anal Canal		Atezolizumab plus Tiraglolumab plus chemotherapy		-		-	
122		NCT04584112	1		Active, not recruiting		Triple Negative Breast Cancer		Tiragolumab plus Atezolizumab plus chemotherapy		-		-	
123		NCT05809895	2		Not yet recruiting		Triple Negative Breast Cancer		ociperlimab + tislelizumab + chemotherapy		Placebo + pembrolizumab + chemotherapy Placebo + tislelizumab + chemotherapy		-	
124		NCT05267054	1/2		Recruiting		B-cell Lymphoma		Ociperlimab plus Tislelizumab Ociperlimab plus Rituximab		-		-	
125		NCT05315713	1/2		Active, not recruiting		Hematological Malignancies		Mosunetuzumab SC plus Tiragolumab Mosunetuzumab SC plus Tiragolumab plus Atezolizumab		-		-	
126		NCT05005442	2		Recruiting		Hematological Malignancies		Pembrolizumab plus vibostolimab		-		-	
127		NCT04150965	1/2		Recruiting		Multiple Myeloma		BMS-986,207 BMS-986,207 plus Pomalidimide plus Dexamethasone		Elotuzumab plus pomalidomide plus dexamethasone Anti-LAG-3 Anti-LAG-3 plus Pomalidimide plus Dexamethasone		-	
128		NCT05289492	1/2		Recruiting		Multiple Myeloma		EOS-448 EOS-448 plus Iberdomide EOS-448 plus Iberdomide plus Dexamethasone		-		-	
129		NCT05061628	1		Recruiting		Mixed Tumors: Advanced Solid Tumor Lymphoma		JS006 plus Toripalimab JS006		-		-	
130		NCT05390528	1/2		Recruiting		Mixed Tumors: Advanced Solid Tumor Lymphoma		HLX301		-		-	
131		NCT04254107	1		Recruiting		Mixed Tumors: Advanced Solid Tumor Lymphoma		SEA-TGT SEA-TGT plus sasanlimab SEA-TGT plus brentuximab vedotin		-		-	
132		NCT04772989	1		Recruiting		Mixed Tumors: Advanced Solid Tumor Lymphoma		AB308 plus Zimberelimab		-		-	

**Abbr.** NSCLC = non-small cell lung cancer; SCLC = small cell lung cancer; ESCC = esophageal squamous cell carcinoma; dual = dual TIGIT and PD-(L)1 inhibitors; P = PD-(L)1 inhibitor; dual + C = dual TIGIT and PD-(L)1 inhibitors combined with chemotherapy; P + C = PD-(L)1 inhibitor combined with chemotherapy; dual + O = dual TIGIT and PD-(L)1 inhibitors combined with other inhibitors

**Table 2:** Anti-TIGIT antibody drugs in clinical development

Drug	Mechanism of action	Sponsor
AZD2936	A bispecific antibody that can target both PD-1 and TIGIT simultaneously	AstraZeneca
HLX301	A bispecific antibody that can target both PD-1 and TIGIT simultaneously	Henlius
HB0036	A bispecific antibody that can target both PD-1 and TIGIT simultaneously	Huaota
IBI321	A bispecific antibody that can target both PD-1 and TIGIT simultaneously	Innovent Biologics
MK-7684 A	A fixed-dose combination formulation composed of Vibostolimab and Pembrolizumab	Merck
Domvanalimab	A Fc-silent humanized IgG1 monoclonal antibody directed against TIGIT	Arcus Biosciences
BMS-986,207	A Fc-silent humanized IgG1 monoclonal antibody directed against TIGIT	Bristol Myers Squibb
BAT6021	An investigational humanized IgG1 monoclonal antibody directed against TIGIT	Bio-thera
PM1021	An investigational humanized IgG1 monoclonal antibody directed against TIGIT	Biotheus
M6223	An investigational humanized IgG1 monoclonal antibody directed against TIGIT	EMD Serono
EOS-448	An investigational humanized IgG1 monoclonal antibody directed against TIGIT	iTeos
Vibostolimab	An investigational humanized IgG1 monoclonal antibody directed against TIGIT	Merck
Etigilimab	An investigational humanized IgG1 monoclonal antibody directed against TIGIT	Mereo
Tiragolumab	An investigational humanized IgG1 monoclonal antibody directed against TIGIT	Roche
Ociperlimab	An investigational humanized IgG1 monoclonal antibody directed against TIGIT	BeiGene
ASP8374	An investigational humanized IgG4 monoclonal antibody directed against TIGIT	Astellas
COM902	An investigational humanized IgG4 monoclonal antibody directed against TIGIT	Compugen
IBI939	An investigational humanized IgG4κ monoclonal antibody directed against TIGIT	Innovent Biologics
JS006	An investigational humanized IgG4κ monoclonal antibody directed against TIGIT	Junshi
SEA-TGT	A nonfucosylated antibody that employs sugar engineered antibody against TIGIT	Seagen Inc
AB308	An investigational humanized IgG1 monoclonal antibody directed against TIGIT	Arcus Biosciences

Although clinical trials on the efficacy and safety of this combination are being conducted on a large scale, a few results are currently available. A phase I study assessed the safety and efficacy of vibostolimab, an anti-TIGIT antibody, alone or combined with pembrolizumab for advanced solid tumors (part A) or NSCLC specifically (part B) [98]. No dose-limiting toxicities occurred at a maximum of 700 mg vibostolimab alone or combined with 200 mg pembrolizumab in 21-day cycles. Treatment-related adverse events (TRAEs) occurred in 56% of patients undergoing the monotherapy and 62% of patients taking the combination therapy in part A, and 56% and 70% of patients with anti-PD-1/PD-L1-refractory NSCLC in part B. Common TRAEs were pruritus, fatigue, rash, and hypoalbuminemia. In terms of efficacy, the ORRs were respectively 0% and 7% in the monotherapy group and combination therapy group in part A, and 26% in anti-PD-1/PD-L1-naïve patients receiving the combination therapy in part B. Another phase I trial evaluated the safety and tolerability of the anti-TIGIT antibody etigilimab alone (phase Ia) or in combination with nivolumab (phase Ib) for locally advanced or metastatic solid tumors [115]. The maximum tolerated dose (MTD) was not reached in both settings (20.0 mg/kg etigilimab or 20.0 mg/kg etigilimab plus 240 mg nivolumab given in 14-day cycles). Among 23 patients who received etigilimab alone, 16 (70%) had TRAEs and 4 (17%) had TRAEs of grade ≥ 3, while 7 (70%) had TRAEs and 2 (20%) had TRAEs of grade ≥ 3 in 10 patients receiving etigilimab plus nivolumab. Rash and pruritus were two of the most frequently observed immune-related AEs in both groups. As for the efficacy, 1 patient had a partial response and 1 patient had an approximately 8-month stable disease in the combination group, while no patient had a partial or complete response in the etigilimab alone setting. Cheng et al. reported that IBI939 plus sintilimab had a manageable safety profile and could improve PFS of patients with metastatic NSCLC and PD-L1 expression ≥ 50% compared with sintilimab alone (median, not reached vs. 6.0 months) [116]. Besides, the phase I AdvanTIG-105 trial demonstrated good tolerance and preliminary antitumor activity in patients with ociperlimab combined tislelizumab group [117, 118]. The ORR of patients in the ociperlimab combined tislelizumab group was 57.5% and in the ociperlimab combined tislelizumab plus pemetrexed group was 54.8%. Patients with higher PD-L1 expression (≥ 25%) had a higher ORR. In total, 77 patients experienced ≥ 1 treatment-TRAEs and 53.6% of them were immune-mediated adverse events. Moreover, 41 patients had ≥ 3 TRAEs and serious TRAEs occurred in 14 patients. Further, in phase I/II KEYMAKER-U02 sub-study 2 A, tri-combination of pembrolizumab plus quavonlimab (an anti-CTLA-4 agent) plus vibostolimab showed an acceptable safety profile as well [119]. Phase Ib/II basket research (ACTIVATE) is investigating the impact of the combination approach on biomarkers as an exploratory objective. There was a decrease in TIGIT^+^ Tregs overall and a rise in the CD8/Treg ratio. NK cells, PD-1^+^ T cells, proliferating CD4 and CD8 effector memory populations, as well as NK cells, were also seen to be on the rise. Moreover, it was observed that IL-2 and IFN-γ production had increased. Additionally, 1 month after therapy, some patients’ ctDNA levels decreased [120]. These results demonstrated that combination therapy of anti-TIGIT and anti-PD-1/PD-L1 treatments has acceptable toxicity and promising antitumor activity.

CITYSCAPE was the first phase II randomized controlled trial to report the efficacy and safety of combining anti-TIGIT and anti-PD-1/PD-L1 agents [24], while two previous phase I trials had reported favorable tolerance and anti-tumor activity of tiragolumab, an anti-TIGIT agent, plus atezolizumab in various cancers before [99]. In the CITYSCAPE trial, 135 patients with NSCLC were assigned to receive tiragolumab or placebo plus atezolizumab. The results revealed significantly prolonged progression-free survival of the tiragolumab plus atezolizumab arm in the total population (HR 0.62; 95% CI 0.42–0.91) and in patients with PD-L1 tumor proportion score (TPS) ≥ 50% (HR 0.29; 95% CI 0.15–0.53). Significantly extended overall survival of the combination group was observed in patients with PD-L1 TPS ≥ 50% (HR 0.23, 95% CI 0.10–0.53) but not in the total population (HR 0.69; 95% CI 0.44–1.07). TRAEs were observed in 82% and 71% of patients in the combination group and the monotherapy group, respectively, and serious TRAEs occurred in 21% and 18%, respectively. More immune-related AEs occurred in the combination group compared with the monotherapy group (76% vs. 47%) but were mostly mild (grade 1–2). Likewise, pruritus, fatigue, asthenia, and rash were some of the common TRAEs. The phase II ARC-7 trial’s [121] findings demonstrated that the combination is superior to zimberelimab alone in terms of ORR and PFS. The median PFS was 10.9 months, and the ORR was 40% among the 45 patients who received treatment with the three drugs (domvanalimab, zimberelimab, and etrumadenant). The median PFS for the 44 patients treated with the two drugs (domvanalimab and zimberelimab) was 12.0 months, however, the 44 patients treated with zimberelimab only had an ORR of 27%. Talking to safety, ≥grade 3 TRAEs occurred in 58% (zimberelimab monotherapy), 47% (two medications), and 52% of the safety population (three drugs). All incidences of rash were grade 1–2, treatable with topical corticosteroids, and more prevalent in patients using three medicines (60%) compared to those taking two drugs (48%) and zimberelimab monotherapy (47%). Nevertheless, the phase III SKYSCRAPER-02 study revealed that treatment of tiragolumab plus atezolizumab plus chemotherapy did not prolong the PFS (HR 1.08; 95% CI 0.89–1.31) and OS (HR 1.02; 95% CI 0.80–1.30) compared with placebo plus atezolizumab plus chemotherapy in patients with extensive-stage SCLC, suggesting that there may be heterogeneity of efficacy of anti-TIGIT plus anti-PD-1/PD-L1 in different cancers [122]. Present research predominantly lies in the recruitment phase whereby only several trials centering on solid tumors yield outcomes. Table 1 outlines 9 clinical trials concerning hematological malignancies, but there is currently no outcome on the TIGIT and PD-1 or PD-L1 combination therapy. We look forward to the findings of more prospective clinical studies.

Taken together, anti-TIGIT and anti-PD-1/PD-L1 combination therapy has shown a favorable safety profile and better antitumor activity along with better survival benefits compared with anti-PD-1/PD-L1 therapy alone, in line with preclinical evidence. Additionally, several new drugs based on the anti-TIGIT and anti-PD-1/PD-L1 combination, such as AZD2936 and MK-7684 A, and novel combination strategies, such as anti-TIGIT plus anti-PD-1/PD-L1 agents plus chemotherapy or chemoradiotherapy, are being evaluated clinically (Table 1). These advances are expected to expand the benefits of the anti-TIGIT and anti-PD-1/PD-L1 combination for cancer patients.

## A prospective on TIGIT blockade therapeutic strategies

Recent studies suggest that TIGIT blockades and radiotherapy (RT) may have a synergistic relationship, although TIGIT and RT are mechanically two different approaches to cancer treatment.

RT can induce an immunogenic antitumor response, but it can also create some immunosuppressive barriers depending on the fractionation protocols employed. For example, 8 Gy*3f and 16.4 Gy*1f protocols induce a lymphoid response (CD8^+^ T cells, Tregs), while the 2 Gy*18f protocol induces a myeloid response (MDSCs, M2 phenotype tumor-associated macrophages) [123]. CD8 T cells secretion of granzyme B was found to be increased by the 8 Gy*3f protocol. And tumor cells showed moderately increased expression of PD-L1 across all fractionation protocols, but most durably with the 2 Gy*18f protocol. While TIGIT expression by CD8^+^ T-cells increased with the 8 Gy*3f protocol and decreased with 2 Gy*18f [123]. Grapin et al. proved that the combination of anti-TIGIT, anti-PD-L1, and 8 Gy*3f (9/10 Complete response, CR) protocol was the most effective treatment strategy [123]. Compared to the 2 Gy*18f radiotherapy alone group, mean tumor volume was significantly lower in the combination of 2 Gy*18f and dual ICI group (p = 0.04). However, the combination of 2 Gy*18f and dual ICI group (7/12 CR) did not outperform than anti-PD-L1 monotherapy combined 2 Gy*18f group (8/12 CR).

Notably, when total radiation of 36 Gy is divided into 3*12Gy, the combination of radiotherapy and anti-TIGIT slowed down primary tumor growth and led to a favorable survival benefit, but this was not observed in secondary tumors [124]. However, low-dose radiation delivered to secondary tumors can reduce the expression of TIGIT receptors in the tumor microenvironment (TME) and contribute to the abscopal response [124].

Moreover, Zhao’s work has demonstrated that combining radiotherapy with anti-TIGIT therapy could slow down primary tumor growth and provide survival benefits. They proved that this combination could stimulate CD8^+^ T cell responses and enhance local accumulation and modulate cytokine production of DCs by blocking the TIGIT/CD155 axis [125]. In addition, the therapeutic response of cancer patients to RT and anti-TIGIT treatment may be strengthened by using Flt3L to boost CD103^+^ DCs at the tumor site.

The findings of Hu et al. provide significant support for the enhancement of effectiveness and validity of combining radiation with concurrent TIGIT and PD-1 inhibitors by nanoparticle [126]. In his investigation, 12 Gy of radiation was administered to the primary tumors. Additionally, in around 30% of the anti-PD1-resistant lung cancer model mice, this nanoparticle-mediated combination treatment may result in the elimination of primary and secondary tumors.

It should be noted that this area of research is still in the early stages, and further studies are needed to fully understand the potential synergistic relationship between TIGIT-targeted immunotherapy and radiotherapy. Nevertheless, the potential synergistic relationship between these two treatments represents a promising new avenue for cancer treatment, and ongoing research will shed lighter on this topic.

## Conclusions

Co-inhibition of TIGIT and PD-1/PD-L1 could synergistically elicit tumor rejection and has been approved in clinical trials, offering a new option for cancer immunotherapy. Although the optimal combination strategy and patient selection criteria are still being investigated, this approach represents a promising avenue for developing more effective cancer immunotherapies. Future research should focus on optimizing treatment regimens to improve patient outcomes and identifying biomarkers to predict response to these therapies. Overall, TIGIT and PD-1/PD-L1 inhibitors hold great potential for enhancing the efficacy of cancer immunotherapies and improving patient outcomes.

## Data Availability

Not applicable.

## Abbreviations

APCs: Antigen-presenting cells
ALL: Acute lymphoblastic leukemia
AML: Acute myeloid leukemia
CTLA-4: Cytotoxic T-lymphocyte-associated protein 4
CLL: Chronic lymphocytic leukemia
DC: Dendritic cell
DNAM-1: DNAX accessory molecule-1 (DNAM-1)
Grb2: Growth factor receptor-bound protein 2
HIF1-α: Hypoxia-inducible factor 1-α
ICAM-1: intracellular adhesion molecule 1
ICIs: Immune checkpoint inhibitors
ITIM: Immunoreceptor tyrosine-based inhibitory motifs
ITSMs: Immunoreceptor tyrosine-based switch motifs
LAG-3: Lymphocyte-activation gene 3 protein
LFA-1: Lymphocyte function-associated antigen 1
MCL: Mantle cell lymphoma
MTD: Maximum tolerated dose
MAPK: Mitogen-activated protein kinases
MDSCs: Myeloid-derived suppressor cells
MM: Multiple myeloma
MSS: Microsatellite-stable
NK: Natural killer
NSCLC: Non-small cell lung cancer
NF-κB: Nuclear factor kappa-light-chain-enhancer of activated B cells
ORRs: Objective response rates
OS: Overall survival
PI3K: Phosphoinositide 3-kinase
PVR: Poliovirus receptor
PD-1: Programmed cell death protein 1
PD-L1: Programmed Cell Death-Ligand 1
PFS: Progression free survival
Tregs: Regulatory T cells
SHIP1: SH domain-containing inositol-5-phosphatase
TCR: T cell receptor
TIGIT: T cell immunoreceptor with Ig and ITIM domains
TRAEs: Treatment-related adverse events
VISTA: V domain immunoglobulin suppressor of T cell activation
