# Correction: Co-inhibition of TIGIT and PD-1/PD-L1 in Cancer Immunotherapy: Mechanisms and Clinical Trials

**DOI:** 10.1186/s12943-023-01812-z

**Published:** 2023-06-27

**Authors:** Xianjing Chu, Wentao Tian, Ziqi Wang, Jing Zhang, Rongrong Zhou

**Affiliations:** 1 Department of Oncology, Xiangya Hospital, Central South University, No. 87 Xiangya Road, Kaifu District, Changsha, 410008 China; 2 Present Address: Xiangya Lung Cancer Center, Xiangya Hospital, Central South University, Changsha, 410008 China; 3 National Clinical Research Center for Geriatric Disorders, Xiangya Hospital, Central South University, Hunan Province, Changsha, 410008 People’s Republic of China

**Correction: Mol Cancer 22, 93 (2023)**

**https://doi.org/10.1186/s12943-023-01800-3**

Following publication of the original article [1], the author reported that in the submitted version of their manuscript and the proofed version of their paper they explicitly stated that Rongrong Zhou and Jing Zhang were co-corresponding authors, although they were unable to add another corresponding author during proofing. The correct co-corresponding authorship has now been emphasized in the author list of this correction note article.

Furthermore, the error of Fig. 2, which is apparently an editing error, as per checking, it needs to be corrected to maintain the conscientiousness of their publication as well as Molecular Cancer. The correct figure is given below.

**Fig. 2:**
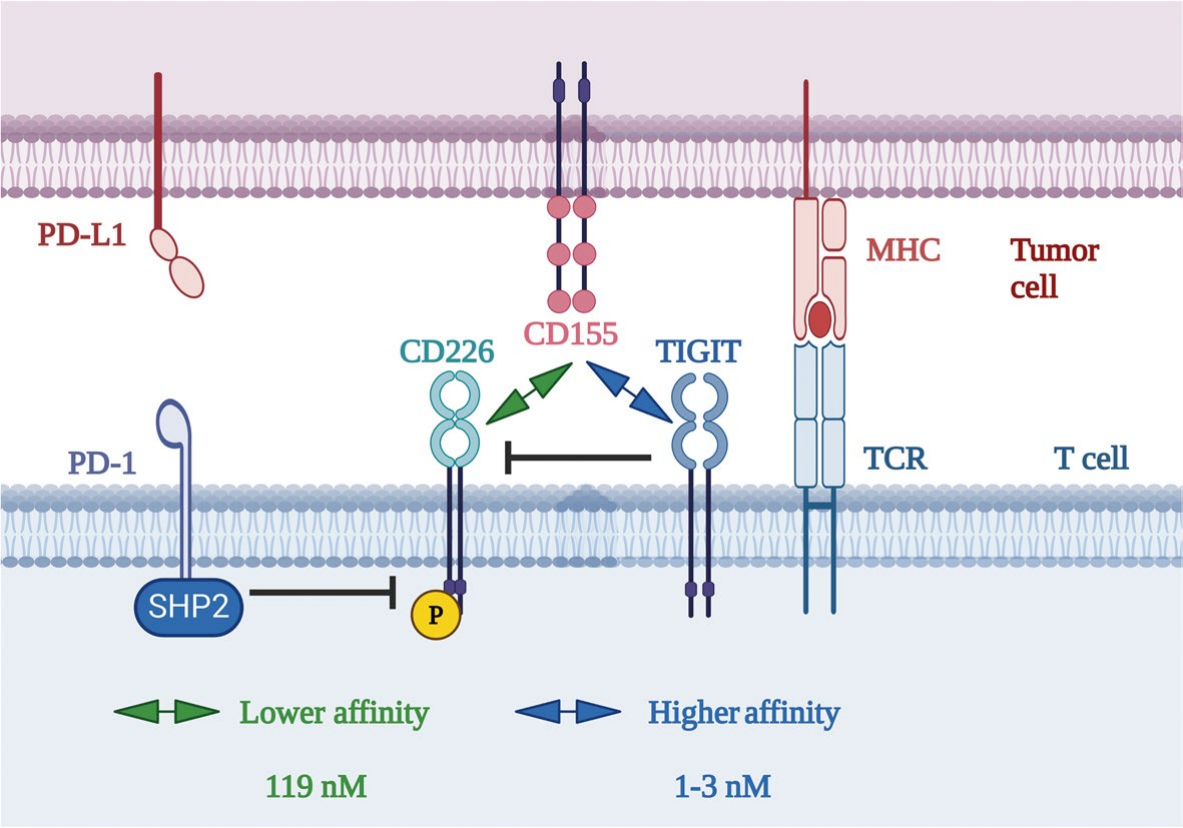
Mechanism of co-inhibition by TIGIT and PD-1. The TIGIT/CD226 pathway and the PD-1/PD-L1 pathway have an intersecting crossroad. On the one hand, upon activation by PD-L1, the intracellular domain of PD-1 recruits Shp2 to dephosphorylate CD226, inhibiting the immune activation function of CD226. On the other hand, TIGIT has a higher affinity (dissociation constant 1–3 nM) to CD155 than that of CD226 (dissociation constant 119 nM) [25], thus competitively antagonizes and blocks CD226 homodimerization through its extracellular domain, inhibiting the immune activation function of CD226
